# The Phytochemical Constituents of Medicinal Plants for the Treatment of Chronic Inflammation

**DOI:** 10.3390/biom13081162

**Published:** 2023-07-25

**Authors:** Young-Joo Kim, Ki Sung Kang

**Affiliations:** 1 Natural Product Research Center, Korea Institute of Science and Technology, Gangneung 25451, Republic of Korea; yjkim7801@kist.re.kr; 2 College of Korean Medicine, Gachon University, Seongnam 13120, Republic of Korea

Prolonged exposure to inflammatory mediators can lead to tissue damage, fibrosis, angiogenesis, and altered cellular metabolism. The sustained presence of inflammatory signals can disrupt normal physiological processes and contribute to the development and progression of various diseases, exacerbating symptoms and compromising overall health. Chronic inflammation can have far-reaching consequences on tissue homeostasis and organ function and is a major risk factor for numerous diseases, including arthritis, cardiovascular disorders, stroke, cancer, and diabetes [1,2,3]. Conventional treatments for chronic inflammation, such as nonsteroidal anti-inflammatory drugs (NSAIDs) and corticosteroids, can have limitations and side effects. Therefore, researchers need to explore new, safe, and effective alternative treatments for chronic inflammation.

Plant-derived phytochemicals have emerged as novel agents for protecting against chronic disorders. As phytochemicals are so diverse, they cover a wide spectrum of therapeutic indications against various inflammation-related diseases, such as cancer, inflammation, cardiovascular, rheumatoid, autoimmune, and neurological disease, and have been a productive source of lead compounds for the development of novel medications [4,5]. Harnessing the potential of phytochemical constituents from medicinal plants offers a promising new class of treatment for chronic inflammation [6]. These natural products provide a diverse range of anti-inflammatory mechanisms and demonstrate excellent safety profiles. Further research is needed to explore their efficacy, optimize dosage regimens, and identify potential drug interactions. Embracing the rich phytochemical repertoire of medicinal plants could pave the way for the development of novel and effective therapies for chronic inflammatory diseases, improving the quality of life for countless individuals worldwide. The multidimensional medicinal approach has been successfully applied through the use of herbal medicines and traditional Chinese medicines (TCM). So Shiho Tang is a well-known TCM (Xiao Chai Hu Tang in Chinese) composed of seven herbs, and is traditionally prescribed to treat various viral infections and inflammatory disorders. In vivo tests of SSHT document its wide range of effects on cancer, fibrosis, inflammation, and several metabolic disorders [7,8].

Recently, efforts to understand the disease by integrating conventional molecular biological experiments, bio-big-data analysis, and AI technologies have increased rapidly. In order to understand the polypharmacological effects of multicomponents, a large amount of time and effort is required, and has only been spent on conventional experiments [9,10]. Therefore, it is possible that a more integrated approach is required to develop a deeper understanding of the disease and better treatments for it.

Despite these beneficial effects and the various studies that have been conducted, quality control (QC) continues to present a problem in ensuring consistent efficacy, as natural products contain a variety of ingredients. Chemical quality control (QC) refers to the assessment of the chemical composition and purity of a drug or herbal medicine [11]. Although chemical QC is important for ensuring the safety and efficacy of drugs, it has some limitations. The chemical analysis does not always reflect biological activity: a compound may be present in high concentrations in a sample, but it may not be the active ingredient responsible for the biological effects of the drug.

To overcome the current QC limitations, biological QC can be performed in addition to chemical QC. Biological QC refers to the assessment of the biological activity of a drug or herbal medicine using in vitro or in vivo methods. It can provide a more accurate assessment of the biological activity of a drug and can help detect potential contaminants or adulterants that may not be detected through chemical analysis. Therefore, biological QC is an important complement to chemical QC in ensuring the safety and efficacy of drugs and herbal medicines.

This Special Issue aims to investigate the phytochemicals used for treating inflammation-related diseases through combinatorial strategies which target multiple mechanisms, such as increasing anti-inflammatory efficacy and triggering a response in innate immune systems which control chromosome stability [12], as these may offer a better chance of discovering a clinically meaningful treatment.

## Data Availability

Not applicable.
